# Should we feed back research results in the midst of a study?

**DOI:** 10.1186/1748-5908-7-87

**Published:** 2012-09-13

**Authors:** Carole A Estabrooks, Gary F Teare, Peter G Norton

**Affiliations:** 1 Faculty of Nursing, University of Alberta, Edmonton, Alberta, T6G 1C9, Canada; 2 Quality Measurement and Analysis, Saskatchewan Health Quality Council, Saskatoon, Saskatchewan, S7N 3R2, Canada; 3 Faculty of Medicine, University of Calgary, Calgary, Alberta, T2N 4N1, Canada

## Abstract

**Background:**

This report is an introduction to a series of three research papers that describe the evolution of the approaches taken by the Translating Research in Elder Care (TREC) research team during its first four years to feed back the research findings to study participants. TREC is an observational multi-method health services research project underway in 36 nursing homes in the prairie provinces of Canada. TREC has actively involved decision makers from the sector in all stages from initial planning, through data collection to dissemination activities. However, it was not planned as a fully integrated knowledge translation project. These three papers describe our progress towards fully integrated knowledge translation—with respect to timely and requested feedback processes. The first paper reports on the process and outcomes of creating and evaluating the feedback of research findings to healthcare aides (unregulated health professionals). These aides provide over 80% of the direct care in our sample and actively requested the feedback as a condition of their continued cooperation in the data acquisition process. The second paper describes feedback from nursing home administrators on preliminary research findings (a facility annual report) and evaluation of the reports’ utility. The third paper discusses an approach to providing a more in-depth form of feedback (expanded feedback report) at one of the TREC nursing homes.

**Findings:**

Survey and interview feedback from healthcare aides is presented in the first paper. Overall, healthcare aides’ opinions about presentation of the feedback report and the understand ability, usability, and usefulness of the content were positive. The second paper describes the use of telephone interviews with facility administrators and indicates that the majority of contextual areas (*e.g.*, staff job satisfaction) addressed in facility annual report to be useful, meaningful, and understandable. More than one-half of the administrators would have liked to have received information on additional areas. The third paper explores how a case study that examined how involvement with the TREC study influenced management and staff at one of the TREC nursing homes. The importance of understanding organizational routines and the impact of corporate restructuring were key themes emerging from the case study. In addition, the Director of Care suggested changes to the structure and format of the feedback report that would have improved its usefulness.

**Conclusions:**

We believe that these findings will inform others undertaking integrated knowledge translation activities and will encourage others to become more engaged in feedback processes.

## 

‘Everybody needs feedback, and it’s a heck of a lot cheaper than paying a trainer.’ -Doug Lowenstein

The following three papers describe the evolution of the approaches taken by the Translating Research in Elder Care (TREC) research team during its first four years to feed back the research findings to study participants. TREC is an observational multi-method health services research project underway in the prairie provinces of Canada. Its focus is care delivered in nursing homes, and it was funded through a five-year operating grant from the Canadian Institutes of Health Research (2007 to 2012). TREC is designed to establish the associations between organizational context, bedside knowledge use, work life quality for caregivers, and resident outcomes. The data sources for TREC include direct reports from frontline caregivers and their managers, facility and unit demographic data, and outcome data generated as part of the regular care process using the *interRAI* Resident Assessment Instrument system [1]. The protocols for the project are in the literature and can be consulted for those who wish more knowledge of the research and analysis plans [2-4].

When TREC was funded the researchers agreed to a set of traditional feedback options. First, the 36 participating nursing homes administrators were to receive structured feedback from the data periodically (annually) during the course of the project. Further, participants were given the opportunity to request a final report at the termination of the TREC project in 2012. The timing of both of these was governed by the research team. As the project proceeded, however, it became clear that participants wanted feedback on a more timely and routine basis. Some facilities requested more in-depth feedback than was given in the standard feedback reports we initially generated. As well, the research climate in Canada was changing. In 2006, our national research funding organization adopted a knowledge-to-action framework to guide knowledge translation [5,6]. In 2009, they adopted specific granting mechanisms to encourage both end-of-grant knowledge translation and use of an integrated knowledge translation approach to ensure involvement of knowledge users with researchers throughout the research process [7]. These national efforts have influenced researchers, caregivers, and decision-makers in our country, and clearly had an effect on the TREC research team.

So, from a rather traditional beginning, the TREC research team has undertaken a journey of discovery not only in the study of conditions conducive for knowledge translation, but also of the doing of it as well. The three papers that follow describe in some detail the evolution of our feedback processes and their effects with respect to three distinct audiences. First, we describe the need for feedback to healthcare aides. Healthcare aides are unregulated health professionals who deliver the vast majority of care at the bedside in the Canadian Prairie province nursing home sector. They have largely been ignored in studies of research utilization and knowledge translation. They were invited on two occasions about one year apart to participate in TREC by completing a 30-minute, computer-assisted personal interview that determined their experience of organizational context, quality of their work life, and their use of best practice at the bedside. After the first round of interviews, they informed our research assistants that they needed to see results of these interviews prior to undertaking a second set. This required a considerable realignment of research team resources. The process and outcomes of creating and evaluating the feedback of research findings to healthcare aides is described in the first paper. The second paper describes an approach to integrated knowledge translation we used in TREC. We provided a standardized and regular form of feedback of preliminary research findings to nursing home administrators over the course of the study and sought their evaluation of its utility. The final paper is a case study at one facility of an approach to providing a more in-depth form of feedback than was provided in the standardized facility reports. The leader of that facility had requested in-depth feedback after receiving her first annual report.

Undertaking these feedback activities clearly challenged our planned resource expenditures and team processes. However, there are other more substantive challenges inherent in such activity. One of the tensions that has largely been ignored in the literature on integrated knowledge translation model is the tension of the traditional research design (in which results are not typically fed back to participants while in progress) and a model where decision-makers and participants more equally balance investigators—and demand to see results as they unfold, however preliminary and tentative those results may be. It has been our experience that this tension, regardless of a team’s commitment to integrated knowledge translation, is constantly present and requires open management. Our work is observational, and thus the stringent guidelines of clinical trials were not at play. Nonetheless, we grappled with the possible impact on wave two data collection if we gave participants some wave one data findings. In the end, we believed the benefits far outweighed any possible negative effects. And, of course, altering behaviour is not as simple as providing a little bit of feedback. The issue is more challenging and perhaps complex when decision makers want preliminary results with which to make real-world decisions. Until data are processed, analyzed, and those analyses checked and rechecked and ultimately published in a peer reviewed venue, many researchers are reluctant to stake even a conditional claim on the veracity and thus usability of the findings. Again, it requires an assessment of benefit and possible negative effects. The reality often is, as we have been told by more than one decision-maker, the decision will be made regardless; without the early findings it may well be made with no data at all.

These three papers [8-10] describe our progress towards integrated knowledge translation in some detail, and we think will inform and encourage others undertaking health services research to become more engaged in knowledge translation activities. We believe this is a high priority for medical research and necessary if findings of health services research are to truly influence the way we deliver care in a variety of healthcare settings and to various populations. An integrated approach to knowledge translation is essential in order to maximize the probability that these processes of care are modified in timely and evidence-informed ways and to help researchers stay grounded in the realities of the people and environments they are hoping to influence through their research. It is also essential to establish clear expectations and processes within integrated teams—processes that enable teams to safely navigate the grey zones of feeding data back.

## Abbreviations

TREC: Translating Research in Elder Care.

## Competing interests

The authors declare that they have no competing interests.

## Authors’ contributions

CAE, GFT, and PGN drafted the short report. CAE and PGN conceived of the TREC study and secured funding for the study. All authors read and approved the final manuscript.

## Authors’ information

CAE is Professor, Faculty of Nursing, at the University of Alberta and is the principal investigator for the TREC research program. GFT is Director of Quality Measurement and Analysis, and Adjunct Professor, Department of Community Health and Epidemiology, University of Saskatchewan, Assistant Professor, Department of Health Policy, Management and Evaluation, University of Toronto, and Adjunct Faculty, Institute for Clinical Evaluative Sciences, Toronto, Ontario and is a co-investigator for the TREC research program. PGN is Professor Emeritus, Department of Family Medicine, University of Calgary and is the co-principal investigator for project one of the TREC research program.
